# Bleeding outcomes in thrombocytopenic acute leukemic patients with venous thromboembolism

**DOI:** 10.1002/jha2.90

**Published:** 2020-09-02

**Authors:** Nathaniel R. Wilson, Maliha Khan, Travis M. Cox, Mohammed Nassif, Wei Qiao, Naveen Garg, Fleur M. Aung, Thein Hlaing Oo, Cristhiam M. Rojas‐Hernandez

**Affiliations:** ^1^ Department of Internal Medicine The University of Texas Health Science Center at Houston Houston Texas; ^2^ Department of Hematology and Oncology The University of Arkansas for Medical Sciences Little Rock Arkansas; ^3^ Department of Hematology and Oncology The University of Texas Health Science Center San Antonio San Antonio Texas; ^4^ Department of Pediatrics – Research Resource Office Baylor College of Medicine Houston Texas; ^5^ Department of Biostatistics The University of Texas M.D. Anderson Cancer Center Houston Texas; ^6^ Department of Diagnostic Radiology The University of Texas M.D. Anderson Cancer Center Houston Texas; ^7^ Department of Laboratory Medicine The University of Texas M.D. Anderson Cancer Center Houston Texas; ^8^ Section of Benign Hematology The University of Texas M.D. Anderson Cancer Center Houston Texas

**Keywords:** acute leukemia, anticoagulation, cancer, platelet count, thrombocytopenia, thrombosis

## Abstract

Cancer‐associated thrombosis in acute leukemia patients with severe thrombocytopenia (platelets ≤50 × 10^9^/L) poses a management challenge due to competing risks of bleeding and recurrent thrombosis. A retrospective analysis was conducted to determine the occurrence of clinically relevant bleeding (CRB) rates during treatment for acute venous thromboembolic events (VTE) in thrombocytopenic acute leukemic patients. A cohort of 74 patients were subgrouped into three VTE‐treatment interventions: anticoagulation (*n* = 24), inferior vena cava filter placement (*n* = 22), and observation (*n* = 28). Multivariate analysis found a significant correlation between CRB occurrence and quantity of overall blood transfusions, chemotherapy administration, and relapsed leukemia presentation. There was no difference in the occurrence of CRB between VTE‐treatment subgroups, regardless of initial platelet count at the time of VTE diagnosis. Regarding the hematologic parameters, only the velocity of the platelet count recovery was associated with the risk of bleeding. From this analysis, it appears the trajectory of the platelet count and the factors associated with a slower recovery of it, are the main determinants for the occurrence of hemorrhagic complications during VTE treatment in acute leukemia.

## 1 INTRODUCTION

Compared to patients without malignancy, cancer patients carry an increased risk of both bleeding events, as well as venous thromboembolic events (VTE) during the course of their disease and treatment [1, 2]. Bleeding in those patients can occur as a result of many intrinsic and extrinsic factors including anemia, thrombocytopenia, vascular integrity, coagulopathy, malignancy, chemotherapy, and infection [3]. The risk of developing a VTE in patients with malignancy is seven times higher than that of the general population; 28 times higher in patients with hematologic cancers [4]. Patients with hematologic malignancy often develop thrombosis as a result of a combination of multiple characteristics such as tumor type, obesity, immobility, complete blood count variables (ie, platelets, hemoglobin, leukocytes), central venous device use, and antineoplastic chemotherapy [5]. It is estimated that 20 to 30% of VTE are associated with malignancy [6] and are one of the leading contributors to mortality in cancer patients [7]. Incidence of VTE in patients with acute leukemia has been recorded between 2% and 12%, often within the first month of leukemia diagnosis [8]. Management of cancer‐associated thrombosis (CAT) is therefore especially prudent in this population. Many patients with hematologic malignancies also frequently develop severe thrombocytopenia (platelets ≤50 × 10^9^/L) during the course of their disease and treatment [9]. In thrombocytopenic patients, platelet count has an imprecise association with increased risk of bleeding. In the PLADO trial, the risk of bleeding was elevated in those with platelet counts ≤5 × 10^9^/L compared to those with platelet counts ≥81 × 10^9^/L, although there was otherwise no clear correlation of decreased bleeding risk with increased platelet counts [10]. Though thrombocytopenia may be associated with bleeding, there is no platelet count threshold at which the risk of bleeding cannot be accounted for [3]. The rates of bleeding in prior studies of patients with CAT and prolonged thrombocytopenia have ranged between 7% and 33% [11, 12]. Prolonged thrombocytopenia for greater than 30 days in patients with CAT not only correlates with an increased risk of bleeding but has also been associated with increased risk of recurrent VTE [6, 12]. Thrombosis and bleeding events negatively impact the quality of life of patients with malignancy and can possibly postpone or halt treatment for their cancer. Development of CAT in patients with thrombocytopenia therefore poses a unique management challenge due to competing risks of bleeding events and recurrent thrombosis.

## 2 MATERIALS AND METHODS

Patients with acute leukemia admitted to the University of Texas M.D. Anderson Cancer Center (MDACC) between January 2002 and March 2016 who developed CAT were retrospectively evaluated through medical chart review. The terms leukemia, deep vein thrombosis (DVT), and pulmonary embolism (PE) were used to search the data. Included patients were older than 17 years, with proximal lower extremity DVT and/or PE, and thrombocytopenia (platelet levels ≤50 × 10^9^/L) in at least two consecutive measures. A total of 74 patients were analyzed, and basic demographic details, Eastern Cooperative Oncology Group (ECOG) performance status, prior history of VTE, and other comorbidities were collected. Patients were separated and analyzed into three VTE‐treatment interventions by which they were managed: anticoagulation (AC) only (*n* = 24), inferior vena cava filter (IVCF) only (*n* = 22), and observation (*n* = 28). Outcomes analyzed within 12 months from VTE diagnosis included rate and location of VTE recurrence, clinically relevant bleeding (CRB) rate and type of bleeding, and other therapy‐related complications that arose. Details on the thrombosis and survival outcomes of this cohort have been published previously [13]. Computations were carried out in SAS version 9.4.

For this study, we collected daily platelet count measures during the hospitalization for VTE, from the time of diagnosis of VTE and the subsequent 30 days, death outcome or hospital discharge, whichever occurred first.

Patients excluded from the analysis were those with other indications for long‐term antithrombotic therapy at time of VTE diagnosis (ie, atrial fibrillation with congestive heart failure, hypertension, age ≥ 75, diabetes, stroke (CHADS_2_) score >2, antiphospholipid syndrome, or other inherited thrombophilia documented), prior placement of nonretrieved IVCF, and those patients with inadequate documentation or follow‐up data in the electronic medical record to evaluate study outcomes. This study was approved by the MDACC Institutional Review Board.

### 2.1 Main outcome measures

To examine the rates of CRB by the VTE‐treatment intervention group in acute leukemia patients with CAT and thrombocytopenia. Association between the CRB events and clinical and laboratory characteristics was analyzed. Outcomes determined within 12 months from initial VTE diagnosis included recurrent VTE and location, CRB occurrence, location, and other complications of therapy.

### 2.2 Definition and criteria of bleeding

CRB was subcategorized into major bleeding and clinically relevant nonmajor bleeding. Major bleeding (MB) was defined using the International Society on Thrombosis and Haemostasis (ISTH) criteria as hemorrhage leading to a hemoglobin drop of 2 g/dL (1.24 mmol/L) or greater, or resulting in transfusion of two or more units of packed red blood cells (RBCs) or whole blood within 24 hours, and/or bleeding in a vital organ such as intraocular, intracranial, intraspinal, pericardial, retroperitoneal, intra‐articular, or intramuscular with compartment syndrome, and/or fatal bleeding [14]. Clinically relevant nonmajor bleeding (CRNMB) was defined as any sign or symptom of bleeding which did not meet the ISTH criteria for major bleeding and prompted face‐to‐face evaluation, medical intervention, hospitalization, or increased level of medical care [15].

### 2.3 Statistical analysis

**FIGURE 1:**
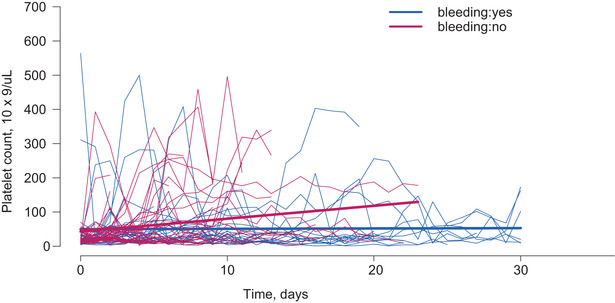
Platelet trends over 30 days by bleeding group. Platelet baseline based on boot strapping OR 1.0106 (*P* = .350, 95% CI 0.9866‐1.0376). Platelet slope based on boot strapping OR 0.8415 (***P*** = .010, 95% CI 0.6841‐0.9989). *OR* odds ratio, *CI* confidence interval. Platelet count in units of 10^9^/L; Time in units of days. Boldface indicates statistical significance

Demographic data and clinical and laboratory parameters were collected (Table 1), and bleeding events retrospectively captured (Table 2). Multivariate regression models compared the association between CRB and multiple clinical variables (Table 3) using Fisher's exact test (two tail) and Wilcoxon rank sum tests. Platelet changes over time stratified by bleeding group were demonstrated using a spaghetti plot along with a smoothing curve (Figure 1). The linear mixed model was used to obtain the subject‐specific platelet trajectory estimation. The final logistic regression for bleeding was built including subject‐specific platelet trajectory estimations and relation to clinical and laboratory factors (Tables 1, 2, 3) based on backward model selection. In order to obtain the correct standard errors for odds ratio estimates (eg, platelet trajectory and platelet baseline) in the final model, the bootstrapping procedure was used (random samples = 1000).

**TABLE 1:** Demographic, clinical, and laboratory profiles of patients

Variable	Total *n* = 74 (%)	Group 1: anticoagulation *n* = 24 (%)	Group 2: IVC filter *n* = 22 (%)	Group 3: observation *n* = 28 (%)	*P*‐value
Age (years) median (minimum, maximum)	54.65 (19, 87)	60 (21, 80)	56 (22, 79)	59 (19, 87)	.80
Gender					
Female	30 (41)	9 (38)	10 (46)	11 (39)	.85
Male	44 (59)	15 (62)	12 (54)	17 (61)	
ECOG status					
0, 1	27 (37)	9 (38)	9 (41)	9 (32)	.72
2	18 (24)	6 (25)	3 (14)	9 (32)	
3, 4	29 (39)	9 (38)	10 (45)	10 (36)	
Platelet count at VTE (× 109/L) Median (minimum, maximum)	20 (2, 50)	28 (4, 48)	26 (8, 50)	15 (2, 46)	**.003**
Prior/concurrent cancer	10 (14)	3 (13)	2 (9)	5 (18)	.76
History of VTE	40 (54)	9 (38)	16 (73)	15 (54)	.06
Histopathology					
ALL	18 (24)				
AML/MDS	56 (76)				
Leukemia status at index event					
De novo	43 (58)	16 (67)	14 (64)	13 (46)	.28
Relapsed/refractory	31 (42)	8 (33)	8 (36)	15 (54)	
Treatment					
Chemotherapy	61 (82)	20 (83)	18 (82)	23 (82)	.99
HSCT	9 (12)	4 (17)	1 (5)	4 (14)	.41
TKI	3 (4)	2 (8)	0	1 (4)	.35
Immunotherapy	14 (19)	6 (25)	2 (9)	6 (21)	.35
VTE event					
PE	21 (28)	10 (41)	3 (14)	8 (29)	.05
DVT	44 (60)	9 (38)	16 (73)	19 (68)	
PE and DVT	9 (12)	5 (21)	3 (14)	1 (4)	

Abbreviations: ALL, acute lymphocytic leukemia; AML, acute myeloid leukemia; DVT, deep venous thrombosis. ECOG, Eastern Cooperative Oncology Group; HSCT, hematopoietic stem cell transplant; MDS, myelodysplastic syndrome; PE, pulmonary embolism; TKI, tyrosine kinase inhibitor; VTE, venous thromboembolism; Boldface indicates statistical significance.

**TABLE 2:** Bleeding severity, location, and transfusion profiles among VTE‐treatment intervention

Variable	Total *n* = 74 (%)	Group 1: anticoagulation *n* = 24 (%)	Group 2: IVC filter *n* = 22 (%)	Group 3: observation *n* = 28 (%)	*P*‐value
Clinically relevant bleeding (CRB)	29 (39.3)	9 (37.5)	10 (45.5)	10 (35.7)	.60
Nonmajor (CRNM)	9 (12.2)	4 (16.7)	2 (9.1)	3 (10.7)	.37
Major	19 (25.7)	4 (16.7)	8 (36.4)	7 (25.0)	
Major and CRNM	1 (1.4)	1 (4.2)	0	0	
Bleeding Location					
DAH/Pulmonary	2 (2.7)				
Gastrointestinal (GI)	2 (2.7)				
Genitourinary (GU)	2 (2.7)				
ICH	6 (8.1)				
Nose/mouth	5 (6.8)				
Nose/GU	1 (1.4)				
Pleural	1 (1.4)				
Retinal	6 (8.1)				
Retinal/nose/GU	1 (1.4)				
Skin/soft tissue	2 (2.7)				
Soft tissue/GI	1 (1.4)				
Transfusions (median units)					
Platelets (minimum, maximum)		20 (0, 69)	11 (0, 135)	12 (0, 59)	.461
RBC (minimum, maximum)		22 (0, 66)	7 (1, 68)	8 (0, 41)	.066
Fresh Frozen Plasma (minimum, maximum)		0 (0, 251)	0 (0, 61)	0 (0, 20)	.7469

Abbreviations: CRNM, clinically relevant nonmajor (bleeding); DAH, diffuse alveolar hemorrhage; ICH, intracranial hemorrhage; RBC red blood cell; VTE, venous thromboembolism.

**TABLE 3:** Clinical and laboratory profiles of those who bled versus those who did not

Variable	Patients who bled *N* = 29 (%)	Patients who did not bleed *N* = 45 (%)	*P*‐value
Age (years) median (minimum, maximum)	53.66 (39, 68)	55.29 (44, 67)	.8296
Gender			.8102
Female	11 (37.9)	19 (42.2)	
Male	18 (62.1)	26 (57.8)	
ECOG status			.7530
0, 1	10 (34.5)	17 (37.8)	
2	9 (31)	9 (20)	
3, 4	10 (34.5)	19 (42.2)	
Other noncancer comorbidities	16 (55.2)	21 (46.7)	.6343
Prior/concurrent cancer	4 (13.8)	6 (13.3)	1.000
History of VTE	14 (48.3)	26 (57.8)	.4789
Histopathology			.5936
ALL	6 (20.7)	12 (26.7)	
AML/MDS	23 (79.3)	33 (73.3)	
Leukemia Status at index event			**.0294**
De novo	12 (41.4)	31 (68.9)	
Relapsed/refractory	17 (58.6)	14 (31.1)	
Treatment			
Chemotherapy	27 (93.1)	34 (77.8)	.0654
HSCT	4 (13.8)	5 (11.1)	.7307
TKI	2 (6.9)	1 (2.2)	.5571
Immunotherapy	4 (13.8)	10 (22.2)	.5447
Recurrent VTE	2 (6.9)	0	.1503
VTE‐treatment intervention			.6035
AC	10 (34.5)	14 (31.1)	
IVCF	10 (34.5)	12 (26.7)	
Observation	9 (31)	19 (42.2)	
Transfusions (median units)			
Platelet (SD)	28 (25.7)	14 (16.5)	**.0012**
RBC (SD)	23 (17.4)	13 (17.1)	**.0017**
FFP (SD)	6 (11.8)	8 (37.4)	.0573
Laboratory profiles (median)			
Serum creatinine (SD)	0.88 (0.51)	0.92 (0.42)	.4085
Fibrinogen (SD)	312 (126)	582 (234)	**.0155**
PT (SD)	16.5 (3.6)	16.2 (2.6)	.8823
INR (SD)	1.48 (0.4)	1.62 (0.84)	.7962
PTT (SD)	34.1 (13.7)	35.4 (9.2)	.2736

Abbreviations: AC, anticoagulation; ALL, acute lymphocytic leukemia; AML, acute myeloid leukemia; ECOG, Eastern Cooperative Oncology Group; FFP, fresh frozen plasma; HSCT, hematopoietic stem cell transplant; INR, international normalized ratio; IVCF, inferior vena cava filter; MDS, myelodysplastic syndrome; PT, prothrombin time; PTT, partial thromboplastin time; RBC, red blood cell; SD, standard deviation; TKI, tyrosine kinase inhibitor; VTE, venous thromboembolism;

Boldface indicates statistical significance.

## 3 RESULTS

There were 74 patients out of a cohort of 2705 with acute leukemia and VTE who met inclusion criteria for analysis. Reasons for exclusion have been described in a previous publication of this same cohort [13]. Demographics, clinical, and laboratory profiles of patients are included in Table 1. Fifty‐six (76%) of patients were diagnosed with acute myeloid leukemia (AML) or myelodysplastic syndrome (MDS), and 18 (24%) of patients were diagnosed with acute lymphoblastic leukemia (ALL). Three separate VTE‐treatment intervention groups were identified: AC only (*n* = 24), IVCF only (*n* = 22), and observation (*n* = 28). Median platelet count among all patients at time of index event was 20 × 10^9^/L. Patients observed for VTE had significantly lower levels of platelets at VTE‐presentation than those treated with AC or IVCF (Table 1). There were only two recurrent VTE events during the study period.

Out of the 74 patients analyzed, 29 (39.2%) suffered CRB event during the study. Of these patients who bled, 9 (31%) had a CRNMB, 19 (65.5%) had MB, and 1 (3.4%) had both MB and CRNMB event. Bleeding severity classification and location, along with blood product transfusion profiles, are listed in Table 2. Characteristics associated with bleeding events in the univariate analysis are described in Table 3. There was no difference between those who bled and those who did not in terms of gender, ECOG performance status, history of prior/concurrent malignancy, history of VTE, or histopathology type. Patients with relapsed or refractory leukemia bled more than those with de novo leukemia (*P* = .0294). The use of AC did not increase the occurrence of CRB (*P* = .6035). Patients who received chemotherapy showed a trend to bleed more than those who did not receive chemotherapy (*P* = .0654). There was no difference in bleeding events between groups based on treatment with hematopoietic stem cell transplantation (HSCT), immunotherapy, or tyrosine kinase inhibitor (TKI) therapy. Patients with CRB received more transfusions of platelets (*P* = .0012), packed red blood cells (pRBC) (*P* = .0017), and fresh frozen plasma (FFP) (*P* = .0573) compared to those who did not have a CRB event. There was no difference in serum creatinine, prothrombin time, or partial thromboplastin time between those who suffered CRB and those who did not. There was a statistically significant difference in the median of fibrinogen levels between groups (*P* = .0155), but all median fibrinogen levels between groups were >300 mg/dL (Table 3).

We evaluated the platelet count change over time by bleeding group within 30 days after the index VTE (Figure 1). Data beyond 30 days for platelet counts were limited on several patients; therefore, only the first 30 days of platelet information was used for estimating platelet trajectory with the linear mixed model based on backward model selection. Baseline platelet count was not a significant factor associated with CRB (*P* = .35). Based on the univariate analysis data, we selected the leukemia status (de novo vs relapsed) and the use of chemotherapy (yes vs no) for the multivariate model based on backward model selection. Additionally, we incorporated the platelet count trajectory for each group (bleeding vs nonbleeding) for the analysis. The final logistic regression for bleeding was built including subject‐specific platelet trajectory estimations and relation to clinical and laboratory factors (Tables 1, 2, 3) based on backward model selection. We found that the rate of change in platelet count was inversely correlated with bleeding chance, such that one unit increase in the slope of platelet trajectory would decrease the bleeding chance by 15.85% (*P* = .01). Chemotherapy administration was associated with an increase in the odds of bleeding by six times (OR 5.9184, 95% CI 1.2715‐46.0940, *P* = .04).

## 4 DISCUSSION

Historically, acute VTE in the general population was treated initially with a brief period of unfractionated heparin (UFH) or low‐molecular weight heparin (LMWH), followed by long‐term oral anticoagulation with a vitamin K antagonist (VKA) [16]. The comparison of Low‐molecular‐weight heparin versus oral anticoagulant therapy for the prevention of recurrent venous thromboembolism in patients with cancer (CLOT) trial in 2003 showed that in cancer patients with CAT, dalteparin (LMWH) decreased VTE recurrence rates at 6 months without an increased risk of bleeding or mortality compared to VKA [17]. There was subsequently a similar but nonstatistically significant trend in VTE reduction in the 2015 CATCH trial, comparing tinzaparin (LMWH) to VKA. Tinzaparin showed a significant decrease in rates of CRNMB compared to VKA, but there was no significant difference in overall mortality or MB [18]. Other studies have supported the use of LMWH in the treatment of VTE in cancer patients [19], and adjusted‐dose LMWH for those with CAT and thrombocytopenia [20], establishing it as the standard of care for this patient population and currently recommended by international guidelines [21].

New clinical trial evidence for the use of direct oral anticoagulants (DOACs) for the management of CAT has been published in the recent years. The anticoagulation therapy in selected cancer patients at risk of recurrence of venous thromboembolism (SELECT‐D) trial [22] and the Hokusai VTE Cancer trial [23] demonstrated noninferiority of DOAC compared to LMWH, but with the cost of increased rates of bleeding, in particular in upper gastrointestinal and genitourinary malignancies [22, 23]. Current clinical practice guidelines from the American Society of Clinical Oncology (ASCO) [24] reflect the findings of the above studies regarding the best method of treatment for CAT. Most recently, data from the ADAM‐VTE and the CARAVAGGIO studies have shown that apixaban does not have a higher risk for bleeding complications (compared to LMWH) when used for CAT [25, 26]. However, patients in all of those trials were excluded if the platelet count was <50‐75 × 10^9^/L and details in outcomes for those who suffered severe thrombocytopenia during the follow‐up period have not been reported. Moreover, acute leukemia populations were underrepresented in those studies. Therefore, there are insufficient data regarding the safety of the use of DOACs in patients with CAT and severe thrombocytopenia and in acute leukemic patients.

Clinicians must balance the opposing risks of recurrent VTE and bleeding when treating patients with CAT. Full‐dose anticoagulation has shown to be a safe and effective method for treating CAT when patients have platelet counts ≥50 × 10^9^/L [11]. Among patients with CAT and severe thrombocytopenia (platelets ≤50 × 10^9^/L), studies have tried management strategies either with dose modification of anticoagulation using half‐dose or prophylactic dose [27], or with full‐dose anticoagulation plus as‐needed support of platelet transfusions [28]. After a systematic review of the literature, the ISTH [29] has offered guidance regarding treatment of patients with CAT and thrombocytopenia. In those patients with high‐risk features (ie, symptomatic, recurrent, or progressive proximal PE or DVT), treatment may be considered with full‐dose anticoagulation (LMWH/UFH) plus transfusion support to maintain platelets greater than 40‐50 × 10^9^/L. In patients with lower risk features (ie, incidental or distal DVT/subsegmental PE) or when platelet transfusion is impractical, a dose modification strategy may be implemented as follows: half‐dose or prophylactic dose anticoagulation (LMWH/UFH) for patients with platelet counts between 25 and 50 × 10^9^/L, and temporarily withholding anticoagulation for those patients with platelet counts <25 × 10^9^/L [29]. Similarly, expert panels endorsed by the Gruppo Italiano Malattie Ematologiche dell'Adulto Working Party on Thrombosis and Haemostasis and the Canadian Expert Consensus have recommended a half‐dose reduction of LMWH/UFH for treatment of acute CAT in patients with platelet counts between 30 and 50 × 10^9^/L, with discontinuation of pharmacologic anticoagulation in platelet counts <30 × 10^9^/L [21, 30]. Previous analysis has shown that appropriate anticoagulation therapy, as described above, is associated with significantly improved overall survival (OS) without an increased rate of CRB events compared to other therapeutic options for CAT. The risk of CRB episodes has been associated with a number of variables, including history of bleeding, infection, coagulopathy, liver or renal dysfunction, tumor type, and method of anticancer therapy [12].

One prior study of thrombocytopenic cancer patients showed that low platelet counts increased the chance of prophylactic platelet transfusions, but not bleeding events. Bleeding risk in this study was increased with antiplatelet and anticoagulant use, prior hematuria or gastrointestinal bleeding, infection, low hemoglobin, and elevated creatinine and urea nitrogen [31]. Another study in patients with CAT undergoing autologous HSCT revealed that there was no difference in either recurrent VTE or CRB events whether anticoagulation was continued or held temporarily during thrombocytopenia. This study also showed that there was no threshold at which higher platelet counts could predict a decreased risk of bleeding among these patients [32].

Regardless of platelet count, these acute leukemia patients have prothrombotic states associated with VTE occurrence. Original analysis of this cohort of patients at our institution found a worse OS trend with both CAT and thrombocytopenia. There was a significant improvement in VTE recurrence and OS in patients treated with anticoagulation, however, suggesting its effectiveness for use in this patient population [14]. Regardless of VTE‐treatment intervention and severity of thrombocytopenia, patients died from similar causes of either recurrent VTE, CRB, progression of cancer, or other causes [14].

Our analysis delved further into examining the risk of recurrent bleeding rates among these patients based on VTE‐treatment intervention, and clinical and laboratory parameters, specifically severity and duration of thrombocytopenia. We followed thrombocytopenic patients’ consecutive platelet counts for up to 30 days, beyond which time, platelet counts were less consistent between patients. Our findings are similar to prior studies which showed that the risk of both VTE recurrence and CRB events were similar between patients with or without significant thrombocytopenia, treated with standard therapy [11, 12, 31, 32]. There was no significant difference in other laboratory parameters (ie, markers of renal function and coagulation) between groups who bled and did not bleed.

In terms of clinical profiles, there was a higher rate of bleeding in patients with refractory or relapsed leukemia at time of study compared to those with de novo leukemia presentation. Patients treated with antineoplastic chemotherapy had higher risk of bleeding than those treated with other anticancer methods. Of our cohort of leukemia patients, most were diagnosed with AML while only a quarter of patients were diagnosed with ALL. This is an important caveat to recognize given that the indication for treatment with antineoplastic chemotherapy, TKI, or immunotherapy varies by histopathology. Of the three patients who received TKI therapy, all had ALL; of the 14 patients who received immunotherapy, eight had AML/MDS and six had ALL; of the 61 patients who received chemotherapy, 46 had AML/MDS and 15 had ALL. There was no statistical difference, however, in bleeding rates by histopathology type. Our study also found a trend that the median unit of platelet transfusions and RBC transfusions were significantly higher in patients who bled than in those who did not. There was a correlation between longer duration of severe thrombocytopenia with more frequent transfusions and increased CRB episodes. All of these findings may suggest a higher likelihood of bleeding in the setting of prolonged thrombocytopenia as a result of chemotherapy administration, which puts patients at higher risk of both bleeding events, and receiving transfusions based on institutional guidelines. Unlike prior studies showing increased risk of bleeding with antiplatelet or anticoagulant use [31], the patients in this analysis who were treated with anticoagulation had no increased risk of developing CRB.

There was a variety of bleeding presentations in our cohort among those who developed CRB, ranging on a spectrum of both major and nonmajor bleeding events (Table 2). Bleeding events recorded during the study period included epistaxis, hematuria, retinal bleeding, upper and lower gastrointestinal bleeding, superficial bruising and internal hematoma formation, pleural bleeding, diffuse alveolar hemorrhage, and intracranial hemorrhage. Management for patients who presented with CRB included immediate cessation of pharmacologic AC, local hemostasis measures such as direct pressure and cauterization when available, and blood product transfusion to support patients with platelets, pRBC, and FFP when indicated. In all cases of CRB in patients being treated with pharmacologic AC, AC was permanently discontinued.

Anticoagulation administration at our institution reflects the most recent society recommendations [29, 30], and we use a LMWH “sliding scale” during thrombocytopenia: subcutaneous enoxaparin 1 mg/kg every 12 hours for platelet count ≥50 × 10^9^/L, 0.5 mg/kg every 12 hours for platelet count 25–49 × 10^9^/L, and suspend anticoagulation for platelet counts <25 × 10^9^/L.

Limitations of this study were retrospective analysis nature, very sick patient population, and relatively small subset of patients. Patients were not randomized to VTE‐treatment subgroup, which was determined based on clinical judgment at the time of each individual case. One third of patients received pharmacologic AC, and the other two‐thirds did not receive any AC for their VTE. Many patients had platelet counts which could not be continually maintained above 50 × 10^9^/L [8], which may have influenced the treating physician in deciding not to use pharmacologic AC at time of VTE. Previous analysis in this cohort showed that patients who were observed without treatment had a significantly lower platelet count than those treated with AC [13]. It also found those treated with AC were more likely in leukemia‐remission state, and IVCF was placed in patients with either DVT or DVT/PE [13]. Therefore, the argument could be made that patients at lower risk for recurrent VTE and CRB were treated with AC, while higher risk patients were managed with either IVCF or observed.

In some cases, patients were not able to be analyzed for longer than 30 days from initial VTE index event for recurrence of VTE, recurrent thrombocytopenia, or development of CRB. This made it difficult to create an accurate longitudinal prediction model for the effect of thrombocytopenia upon CRB risk over a longer period of time. Additionally, we could not analyze the effect of posttransfusion platelet counts on the risk of bleeding. Nevertheless, our data analysis included platelet count trajectories that may be more representative than single and cross‐sectional measures when assessing the impact of platelet count and bleeding in acute leukemia patients [33].

There was a significant correlation between occurrence of CRB and quantity of overall blood product transfusions, chemotherapy usage, and relapsed leukemia presentation. Prolonged duration of thrombocytopenia in refractory patients receiving antineoplastic therapy often require more frequent blood product transfusions, which offers a plausible relationship for higher risk of bleeding. There was an indirect relationship between median fibrinogen level and bleeding rates among patients (although all median fibrinogen levels between groups were >300 mg/dL). There was no difference in the occurrence of CRB between VTE‐treatment subgroups (AC vs IVCF vs observation), regardless of platelet count at time of VTE diagnosis. From this retrospective analysis, it appears that bleeding complication during AC therapy for CAT in severely thrombocytopenic patients with hematologic malignancy is associated with the duration and severity of thrombocytopenia. The AC treatment strategy for CAT in severely thrombocytopenic leukemic patients with reduced‐dose LMWH does not seem to represent an additional risk for bleeding occurrence.

## AUTHORSHIP CONTRIBUTIONS

CMR and THO conceived the study, performed data analysis, reviewed, and approved the final manuscript product. NRW wrote the manuscript. NRW, MK, TMC, MN, WQ, NG, and FMA performed data collection and analysis, reviewed, edited, and approved the final manuscript product.

## DISCLOSURE OF CONFLICTS OF INTEREST

CMR received funding from Daichii Sankyo for clinical research not related to the current manuscript. THO was a site coinvestigator for Daichii Sankyo, Janssen and Janssen, received honoraria from the Medical Education Speakers Network and served on the advisory board of Bristol Myer Squibb, not related to this manuscript. NG is the CEO of Garglet LLC, a radiology informatics software company. NRW, MK, TC, MN, MA, WQ, and FA have nothing to disclose.

## DATA SHARING STATEMENT

The data that support the findings of this study are available on request from the corresponding author. The data are not publicly available due to privacy or ethical restrictions.
